# Do Parent–Child Dyads with Excessive Body Mass Differ from Dyads with Normal Body Mass in Perceptions of Obesogenic Environment?

**DOI:** 10.3390/nu12072149

**Published:** 2020-07-19

**Authors:** Karolina Zarychta, Anna Banik, Ewa Kulis, Monika Boberska, Theda Radtke, Carina K. Y. Chan, Karolina Lobczowska, Aleksandra Luszczynska

**Affiliations:** 1 Wroclaw Faculty of Psychology, SWPS University of Social Sciences and Humanities, 53-238 Wroclaw, Poland; abanik@swps.edu.pl (A.B.); ekulis@swps.edu.pl (E.K.); mboberska@swps.edu.pl (M.B.); klobczowska@swps.edu.pl (K.L.); 2 School of Psychology and Psychotherapy, Witten/Herdecke University, 58456 Witten, Germany; Theda.Radtke@uni-wh.de; 3 School of Psychology and Public Health, La Trobe University, Flora Hill, VIC 3550, Australia; Carina.Chan@latrobe.edu.au; 4 Trauma, Health, & Hazards Center, University of Colorado at Colorado Springs, Colorado Springs, CO 80918, USA

**Keywords:** childhood obesity, parent–child dyads, food availability, advertising, healthy diet, promotion programs

## Abstract

Background: This study addressed differences between parent–child dyads with excessive body mass (overweight or obesity) and dyads with normal body mass in obesity determinants, derived from social-ecological models. It was hypothesized that parents and their 5–11 years-old children with excessive body mass would (1) report lower availability of healthy food at home, (2) perceive fewer school/local community healthy eating promotion programs, (3) report lower persuasive value of food advertising. Methods: Data were collected twice (T1, baseline; T2, 10-month follow-up), including *n* = 129 parent–child dyads with excessive body mass and *n* = 377 parent–child dyads with normal body mass. Self-reported data were collected from parents and children; with body weight and height assessed objectively. General linear models (including analysis of variance with repeated measures) were performed to test the hypotheses. Results: Compared to dyads with normal body mass, dyads of parents and children with excessive body mass perceived lower availability of healthy food at home and fewer healthy eating promotion programs at school/local community (T1 and T2). These effects remained significant after controlling for sociodemographic variables. No significant differences in persuasive value of food advertising were found. Conclusions: Perceptions of availability of healthy food at home and healthy nutrition promotion may be relatively low in parent–child dyads with excessive weight which, in turn, may constitute a risk factor for maintenance of obesity.

## 1. Introduction

The prevalence of overweight and obesity among children has doubled in recent decades, both in developed and developing countries [1,2]. Obesity is often considered as a result of an exposure of children to an unhealthy environment (also called obesogenic environment) and children’s perceptions and responses to it [2,3]. The role of the obesogenic environment and the ways it is perceived are highlighted in several theoretical approaches explaining childhood obesity. For example, according to the ecological model of predictors of childhood obesity [4], characteristics of at-home-environment (e.g., types of food available at home), and out-of-home environment (e.g., community, demographic and societal characteristics, food policies at school or local community, policies regulating food advertising to children, etc.) represent the facets of a broader context, interacting with each other in the development and maintenance of childhood overweight/obesity.

Availability of various types of food at home is often considered a key determinant of children’s nutrition behaviors [4,5]. In turn, unhealthy nutrition (diet low in fruit and vegetable intake, and high in energy-dense food intake) is significantly associated with excessive body mass [6]. Systematic reviews of environmental correlates of obesity-related behaviors in children showed that the availability of healthy food at home was associated with higher children’s fruit and vegetable intake [7,8]. On the other hand, home availability of sugar-sweetened beverages was associated with a higher intake of these products by 8- to 13-year-olds [9], and intake of sweet and savory snacks among 12- to 13-year-old girls [10]. Lower perceived at-home availability of snacks and sweetened beverages was directly associated with lower intake of respective food among 10- to 11-year-olds [11]. Most of the studies, however, accounted only for children’s perceptions of home food availability and did not consider parental perceptions. Parental perceptions may operate together with children’s perceptions of availability, as parents are the key food gatekeepers at home. Furthermore, it is unclear whether children’s and parental perceptions of availability of healthy food differ depending on body mass status of parent and child (normal body mass versus excessive body mass, i.e., overweight or obesity).

It is unclear if parents and their children with normal differ from those with excessive body mass in terms of their perceptions of availability of healthy food at home between. A cross-sectional study comparing 35 families with parents and children with excessive body mass with 47 families with normal body mass indicated that lower vegetable availability (rated by an independent observer) was associated with obesity issues [12]. This study, however, does not clarify how availability was perceived by parents and children. Determining the levels of parental and child perceptions of food availability at homes of families with overweight parents and children may be of practical relevance. Identifying if families differ in perceptions of at-home and out-of-home environment (depending on body mass status of family members) would allow designing more effective obesity prevention programs, targeting the general population, and family treatment programs for parents and children with excessive body mass [13].

Children’s healthy nutrition and favorable changes in body mass are also shaped by perceptions of out-of-home environment, such as school and local community promotion of healthy eating which, in turn, may influence both parents’ and children’s behaviors and cognitions related to healthy food intake [4]. The World Health Organization [2] has recommended comprehensive programs promoting the intake of healthy food and a reduction of unhealthy food intake in schools as the key environmental strategies to address childhood obesity. An analysis of the effectiveness of 124 nutrition and physical activity programs indicated that the programs accounting for three settings (community, school, and home) were the most effective in terms of childhood obesity prevention [14]. The target population’s awareness of out-of-home programs promoting healthy nutrition may be a condition for the successful implementation of such programs and their effectiveness [15]. Previous dyadic research has found out that parental perceptions of school and community-based physical-activity promotion programs are related to lower body mass in children [16]. It is unclear, however, whether perceptions of availability of nutrition programs may differ among parents and children with excessive body mass versus normal body mass.

Previous research investigating children’s perception of healthy food environment indicated that those who are 5–9 years old perceive their parents and mass media as the primary source of nutrition information [17]. Thus, at-home availability of healthy food and perceptions of food advertising have been investigated in children as young as 5–9 years old [17,18]. Although teachers are reported by children as the source of information on healthy food, qualitative research did not elicit perceptions of programs at local community or at the school setting as relevant sources of information about health or healthy diet among young children [17]. Therefore, an adequate approach to investigate perceptions of 5–11 years old children may be to focus on at-home availability of food or perceptions of advertising, instead of testing young children’s perceptions of a broader environment (e.g., local community).

In parallel to perceptions of food availability at home and availability of nutrition programs at the local community, perceptions of advertising have been shown to determine children’s nutrition behaviors [19,20]. Food marketing practices are considered an environmental factor that can affect adults’ and children’s beliefs, attitudes, and knowledge about healthy eating, and their body mass [21]. Children’s food decisions are made in an environment where food is extensively advertised to stimulate consumption at home, and where respective types of food are perceived as easily available [22,23]. Compared to children with normal body mass, 4–11-year-olds with excessive body mass had a higher recognition of energy-dense food advertisements [24] or food advertisements in general [25]. On the other hand, research suggested that children who are obese may know less about the persuasive value of food advertising [20]. Parents and their perceptions of advertising may play a role in modifying the impact of food advertising on children, e.g., through explaining the nature and selling intent of advertising [26,27]. To date, research has not clarified whether parental and child perceptions of advertising (e.g., its persuasiveness) of food may differ between families with parent and child with excessive body mass, compared to those with normal body mass.

This study investigated the differences between parent–child dyads with excessive body mass and parent–child dyads with normal body mass in terms of: perceptions of at-home environment (availability of healthy food at home) and out-of-home environment (perceptions of school and local community promotion of healthy eating, perceptions of advertising in terms of its persuasiveness). In particular, it was hypothesized that, compared to parents and children from dyads with normal body mass, parents and their 5–11-year-old children from dyads with excessive body mass would (1) report lower availability of healthy food at their homes, (2) perceive fewer healthy eating promotion programs at schools and local community, (3) report lower persuasive value of food advertising.

Moreover, we explored a 10-month stability of differences in perceptions of at-home and out-of-home environment, testing if any changes over time would occur in parent–child dyads with normal body mass and dyads with excessive body mass. During middle childhood (5–11 years old), children’s perceptions of healthy food environment are influenced by their age and the developmental stage [28] and, in consequence, these perceptions may change over one year. Thus, it was investigated whether children’s perceptions of at-home environment and perceptions of food advertising would change over a 10-month period. Finally, to account for the potential confounding effects of parental education, parental perceived economic status, and the location of the residence [29], the hypothesized effects were controlled for possible sociodemographic covariates.

## 2. Materials and Methods

### 2.1. Participants

Parents (98.6%) or legal guardians (1.4%; henceforth called “parents”) that were the main caregivers in terms of preparing food and time spent with a child were included in the study as well as their 5–11-year-old children. The initially recruited sample included 924 dyads (1848 individuals) consisting of parents and their 5–11-year-old children participating in the measurement at Time 1 (T1, baseline), and 571 dyads (1142 individuals) at Time 2 (T2, 10-month follow-up). Data were collected as a part of a larger study testing parental and child psychosocial determinants of body mass [30,31].

At T1, the majority of parents (*n* = 547, 59.2%) from the initially recruited sample had normal body mass, *n* = 355 (38.4%) had excessive body mass, and *n* = 22 (2.4%) had underweight. Among children, *n* = 617 (66.8%) had normal body mass, *n* = 222 (24.0%) had excessive body mass, and *n* = 85 (9.2%) had underweight after adjusting for age and gender in relation to International Obesity Task Force cut-off points [32]. All participants were Caucasian (as 98% of Poland’s population [33]).

Dyads in which either parent or child had underweight (*n* = 126 dyads) were excluded from further analyses, as the factors underlying underweight were not investigated in this study. The remaining sample was divided into the subgroups of parents and children recruited form dyads with a specific body mass composition (e.g., both parent and child with excessive body mass). The dyads with the mixed body mass composition (e.g., consisting of a parent with obesity and a child with normal body mass) were included in additional analyses only (see Appendix A). The mixed body mass composition dyads included *n* = 193 dyads with parents with excessive body mass and children with normal body mass as well as *n* = 88 dyads with parents with normal body mass and children with excessive body mass.

The main analyzed sample consisted of *N* = 506 parent–child dyads (1012 individuals), including *n* = 129 dyads with parent and child who both had excessive body mass and *n* = 377 dyads with parent and child who both had normal body mass. In this study, we use the term ‘dyads’, to highlight the specificity of the subgroup (dyads were not treated as the unit of analysis).

Demographic characteristics of the main analyzed sample (*N* = 506 dyads) and both subsamples (dyads with normal body mass, dyads with excessive body mass), as well as the differences between the subsamples are presented in Table A1.

### 2.2. Procedure

The convenience sample was recruited in 26 locations in six administrative regions of Poland representing three levels of the mean household income (the average, below the average, above the average [33]). Data from parents and children were collected at schools, in general practitioners’ offices, or at participants’ homes. In cases where a school was the location of data collection, dyads with children attending classes in the respective school (but also dyads with children attending other schools but living in the local community) were invited and recruited. In cases of dyads recruited via general practitioners’ offices, children attended various schools in the respective city/town.

Study personnel informed participants about the research aims and procedure. Parents provided informed consent (with respect to their own and their child’s participation) and the child gave assent to participate in the study. Afterward, de-identified codes were assigned to participants to secure their anonymity across the measurement points. Younger children (aged 5–8) were interviewed using a structured interview while older children (aged 9–11) completed a questionnaire. Parents completed the questionnaires separately from children (e.g., in a different room). Participants’ body mass and height were measured with certified scales and rods at both T1 and T2.

At both T1 and T2 (10 months later), parents provided their data referring to their perceptions of at-home environment (perceptions of availability of healthy food at home) and out-of-home environment (perceptions of school and local community promotion of healthy eating, perceptions of advertising in terms of its persuasiveness). Children provided their data with reference to perceptions of availability of healthy food at home, and perceptions of food advertising at both T1 and T2. During the follow-up, study personnel revisited the study sites after contacting parents by phone. The attrition occurred due to parental decisions to change the school/general practitioner or parental or children’s decisions to discontinue their participation at T2.

The study was approved by the Internal Review Board at SWPS University of Social Sciences and Humanities, Wroclaw, Poland. All procedures were in accordance with the ethical standards of the institutional research ethics committee and in line with the 1964 Helsinki declaration and its later amendments.

### 2.3. Materials

Variables measured in both members of the dyad were assessed with the same measures [34]. The feasibility of item-wording for children was tested in a pilot study with *n* = 18 children (aged 5–11 years old) and found to be satisfactory.

#### 2.3.1. Parental and Child Perceptions of Availability of Healthy Food at Home (T1 and T2)

Parental and child perceptions of availability of healthy food at home were measured by four items, each based on Comprehensive Feeding Practices Questionnaire (CFPQ [35]), e.g., “Most of the food I keep in the house is healthy”). Participants were provided with a definition of healthy food, indicating that healthy meals include a lot of raw fruit and vegetable but limited amounts of products with added sugar or salt (e.g., limited amount of salty or sweet snacks) and a limited amount highly processed products (e.g., sausage, cheese). The responses ranged from 1 (*definitely not*) to 4 (*definitely yes*). Higher scores represent higher levels of parental or child perception of availability of healthy food at home. The mean item score for parents was *M* = 3.05, *SD* = 0.40, *α* = 0.54 at T1 and *M* = 3.07, *SD* = 0.32, *α* = 0.56 at T2; for children it was *M* = 2.84, *SD* = 0.44, *α* = 0.56 at T1 and *M* = 3.07, *SD* = 0.32, *α* = 0.58 at T2. Although the reliability coefficients are relatively low, they may be considered acceptable considering the scales had only 4 items [36].

#### 2.3.2. Parental Perceptions of School and Local Community Promotion of Healthy Eating (T1 and T2)

Parental perceptions of school and local community promotion of healthy eating was measured with two items based on Stok et al. [37]: “At school my child draws attention to the issues of healthy revival” and “A lot of things are being done to help me and my child to eat more healthily”. The responses ranged from 1 (*definitely not*) to 4 (*definitely yes*). Higher scores represent a higher level of parental perceptions of school and local community promotion of healthy eating. The mean item score was *M* = 2.80, *SD* = 0.67, *r*_s_ = 0.58 at T1 and *M* = 2.81, *SD* = 0.53, *r_s_* = 0.51 at T2.

#### 2.3.3. Parental and Child Perceptions of Food Advertising (T1 and T2)

Parental and child perceptions of food advertising (its persuasive value) were measured with one item each based on Food Advertising Questionnaire [38], e.g., “Advertising makes food products seem better than they really are”. The responses ranged from 1 (*definitely not*) to 4 (*definitely yes*). The higher scores represent the higher levels of parental or child knowledge of persuasive value of food advertising. The item score for parents was *M* = 2.42, *SD* = 0.98 at T1 and *M* = 2.51, *SD* = 1.06 at T2; for children it was *M* = 2.57, *SD* = 0.80 at T1, and *M* = 2.51, *SD* = 0.80 at T2.

#### 2.3.4. Body Weight and Height (T1)

Child and parental body weight and height were assessed with standard medically approved telescopic height measuring rods and floor scales (scale type: BF-100 or BF-25; Beurer, Germany, measurement error <5%). For children, age and gender specific BMI z-score values were calculated with WHO AnthroPlus macro [39]. For parents, BMI was calculated using body weight and height: BMI = weight (kg)/height^2^ (m^2^).

#### 2.3.5. Sociodemographic Variables (T1)

Parental education was measured with a 5-point scale, ranging from 1 to 5 (primary, uncompleted secondary/vocational, secondary, ≤3 years of higher education, ≥4 years of higher education). Higher scores indicate higher education. Perceived economic status was assessed with one item, “Compared to the average economic situation of the family in the country, how would you rate the economic situation of your family”, with responses ranging from 1 (*much below the average*) to 5 (*much above the average*). Higher scores indicate a higher economic status. The size of the place of residence was assessed with one question, “What is the number of inhabitants in the city/town/village where your family lives” with 4-item response scale (<10,000 inhabitants; between 10,000 and 100,000 inhabitants; between 100,000 and 500,000 inhabitants; >500,000 inhabitants). Higher scores indicate a larger population living in the place of residence.

### 2.4. Data Analysis

Assuming effect sizes of *f* = 0.15, power of 0.95, Type I error rate of 0.05, the sample size was estimated with G*Power calculator [40]. The estimation indicated that at least 120 dyads per a subsample should be recruited, if the analyses would be conducted accounting for potential covariates. Results yielding a *p*-value of 0.05 were considered to be statistically significant. Missing data were accounted for by using the full information maximum likelihood procedure performed in IBM AMOS 25 [41]. All analyses were conducted with SPSS version 25. Analyses of variance were performed to test the differences in parental and/or child perceptions of at-home (perceptions of availability of healthy food at home) and out-of-home environment (perceptions of school and local community promotion of healthy eating, perceptions of food advertising) between parent–child dyads with excessive body mass and dyads with normal body mass. General linear models with repeated measures were performed to test: (1) the time effects on perceptions of at-home and out-of-home environment measured at T1 and T2 in parent–child dyads with excessive body mass vs. dyads with normal body mass, as well as (2) the interaction effects of time and the type of subsample (excessive body mass vs. normal body mass dyads). Sensitivity analyses were conducted to test the robustness of findings [42] and to identify if the patterns of effects are similar when accounting for the effects of control variables (the parental education level, the parental perceived economic status, and the size of the place of residence).

## 3. Results

### 3.1. Preliminary Analysis

The differences between parents who participated at both T1 and T2 measurements and those who dropped out were not statistically significant in terms of perceptions of availability of healthy food at home, perceptions of school and local community promotion of healthy eating, perceptions of food advertising, age, BMI, all *Fs* < 2.32, *ps* > 0.129, or gender, *χ*^2^(1) = 2.37, *p* = 0.306. The differences between children who participated at both T1 and T2 measurements and those who dropped out were not statistically significant in terms of perceptions of availability of healthy food at home, perceptions of food advertising, age, or BMI, all *Fs* < 2.36, *ps* > 0.137. However, dyads with boys tended to drop out more often than dyads with girls, *χ*^2^(1) = 3.26, *p* = 0.072.

Parents from dyads with excessive body mass differed from parents from dyads with normal body mass in terms of gender, *χ*^2^(1) = 12.83, *p* = 0.002, education level, and economic status, all *Fs* > 4.41, *ps* < 0.036. Parents in dyads with excessive body mass were more often men, reported a lower level of education, and a lower perceived economic status than parents in dyads with normal body mass. The differences between two types of dyads were not statistically significant in terms of parental and child age, children’s gender, or the size of the residence place. For details see Table A1.

Bivariate correlations between the study variables obtained for the main analyzed sample of *N* = 506 dyads (*N* = 1,012 individuals) are presented in Table A2. At both T1 and T2, healthy food availability and advertisement perceptions reported by parents were positively associated with children’s perceptions of healthy food availability and perceptions of persuasiveness of advertisement. A higher level of parental education was related to higher availability of healthy food reported by children (T1 and T2). A higher level of parental perceived economic status (T1) was positively associated with healthy food availability, reported by parents and children (T1 and T2), and negatively with parental and children’s BMI (T1 and T2).

#### 3.1.1. Differences between Parent–Child Dyads with Excessive and Normal Body Mass: Perceptions of At-Home and Out-of-Home Environment

Compared to parents from dyads with normal body mass, parents from dyads with excessive body mass reported lower availability of healthy food at their homes (T1 and T2) and fewer school and local community promotion of healthy eating (T1 and T2). There were no statistically significant differences between parents from dyads with normal body mass and dyads with excessive body mass in terms of perceptions of persuasiveness of food advertisement (T1 and T2). The respective findings are reported in Table 1.

Compared to children from dyads with normal body mass, children from dyads with overweight/obesity reported lower availability of healthy food at their homes at T1 and T2 (see Table 1). However, there was no statistically significant difference between children from the two types of dyads in terms of perceptions of out-of-home environment (perceptions of persuasiveness of food advertisement at T1 and T2).

The same pattern of differences was found in sensitivity analysis, testing differences in parental and/or child perceptions of at-home (perceptions of availability of healthy food at home) and out-of-home environment (school and local community promotion of healthy eating, perceptions of persuasiveness of food advertising). In sensitivity analyses, parent–child dyads with normal body mass and dyads with excessive body mass were compared when controlling for parental education level, parental perceived economic status, and the size of the place of residence (see Table 1).

The results of additional analyses, comparing the four types of dyads (i.e., the dyads with normal body mass, dyads with excessive body mass, and the two types dyads with mixed body mass composition) are presented in Appendix A. The additional analyses showed only two significant differences, both referring to parental perceptions. Parents from dyads consisting of a parent with excessive body mass and a child with normal body mass reported lower healthy food availability (T1, T2), compared to parents from dyads with normal body mass.

#### 3.1.2. Changes over Time in Perceptions of At-Home and Out-of-Home Environment among Parent–Child Dyads with Normal and Excessive Body Mass

Regarding the changes over the 10-month period, parental and children’s perceptions remained stable over time. Furthermore, all Time x Group interactions were not significant, neither when tested without nor with control variables such as parental education, parental perceived economic status, or the place of residence (see Table A3). These findings suggest that the gap in perceptions of healthy nutrition options in at-home and out-of-home environment did not decrease over time, with families with excessive body mass perceiving a relatively low availability of healthy food and fewer school and local community promotion of healthy eating, controlling for confounding effects of socio-economic variables.

## 4. Discussion

This study examined the differences between parent–child dyads with excessive body mass versus normal body mass in terms of their perceptions of healthy food-promoting environment. The findings support the assumption that perceptions of factors related to at-home environment and out-of-home environment differ, depending on the body mass status [4]. In particular, parents and children from dyads with excessive body mass perceived lower availability of healthy food at home than parents and children from dyads with normal body mass status. Additionally, parents with excessive body mass status reported lower levels of school and local community promotion of healthy eating, compared to parents from dyads with normal body mass. These differences remained significant after controlling for the level of parental education and economic status, and the size of the place of residence. There were no statistically significant differences between parents and children from the two types of dyads (with excessive body mass versus with normal body mass) in terms of perceptions of persuasiveness of food advertisement.

The findings showing differences in perceived home availability of healthy food products are partially in line with the existing evidence [12]. Previous studies, however, used the ratings of external observers to assess availability of fresh vegetable in households [12]. Our study adds to these findings [12], clarifying that healthy food availability at home is observed differently in families with parents and children with overweight/obesity, compared to families with children and parents with normal body mass. Thus, dyads with excessive body mass are at risk of further body mass increase, due to perceptions of low availability of healthy food. As well documented in previous research, low perceived availability of healthy food may be a trigger for unhealthy nutrition habits [8], that in turn determine a further increase of body mass [13].

The present study also showed that parents from dyads with overweight/obesity perceived lower availability of community and school-based healthy nutrition programs. Previous longitudinal research showed that if parents perceive limited promotion of physical activity in local community or schools, then their overweight children gain even more weight [16]. Therefore, families with children and parents with excessive body mass, in which parents report low levels of community and school healthy nutrition programs, may be at risk of a further increase of body mass in children.

The results did not confirm statistically significant differences between the two types of parent–child dyads (with excessive body mass versus with normal body mass) in terms of perceptions of food advertisement. Previous research suggested that children who are obese know less about persuasive value of food advertising [20], yet the number of studies addressing this issue is limited. A lack of statistically significant differences in the present study was observed even when controlling for age, which is among key determinants of child food advertising knowledge and literacy [43]. Previous studies, however, did not account for parental perceptions of persuasiveness of food advertising. In turn, our study showed that the difference between parents from dyads with excessive body mass and parents with normal body mass was not statistically significant in terms of perceptions of persuasiveness of food advertising. It is possible that parents from both types of dyads interacted similarly with their children, for example when explaining the persuasive value of advertising. Parents may use strategies such as mediation, including deliberate comments and judgments about TV commercials, or explaining the nature and purpose of advertising [26,44]. Similarities across dyads in terms of parental strategies may result in a lack of differences in children’s perceptions of persuasiveness of advertising. Future research may also look more carefully into interactions between parental education [29] and parental practices [31] that may jointly predict children’s perceptions of food advertising. Furthermore, perceptions or judgements other than persuasiveness of food advertising may better differentiate between dyads with excessive body mass and those with normal body mass. For example, recognition of logos (higher levels of fast food logos recognition compared to logos of other types of food among children with excessive body mass [24]), or the effect of exposure levels to food adverts on the energy intake in children with excessive body mass [45] were found to differentiate between the children with normal body mass and with overweight/obesity. Yet, the findings of the present study suggest that it may be relevant to account for the parental perceptions as well.

This study has several limitations. Only healthy food availability was assessed, whereas previous research suggested that assessing availability of both healthy and unhealthy food availability is relevant. Fruit and vegetable intake may be inversely associated with availability of unhealthy food; however, at the same time, higher low calorie and nutrient dense food availability was associated with higher child’s intake of sweet and savory snack [18], which may suggest that certain products might be considered as less healthy than the others and that home environments might be healthy in some ways and at the same time unhealthy in another way (e.g., availability of healthy and unhealthy food products might be perceived as high). Future studies should account for perceptions of availability of healthy food and perceptions of availability of unhealthy food. Moreover, only self-reports of food availability were used. Perceived food availability is likely to be a different construct than the actual availability of food at home, and the two are only moderately related [46]. Therefore, the conclusions of the present study should not be generalized to the differences in actual availability of healthy food. A combination of subjective and objective indicators of at-home availability of food (e.g., photographs of food stored in the family’s pantry or scanning food barcodes during grocery shopping) would be preferable [47]. Yet, the feasibility of using objective measures of food intake in large samples is limited. The study did not account for an actual school-based and local community promotion of healthy eating. Using such methods would allow for controlling whether parental and children’s perceptions of at-home or out-of-home environment correspond with the actual presence of policies and programs at schools/communities. Moreover, the single-item measurement of perceptions of persuasiveness of food advertisement may have limited reliability. Future research could consider more complex measures of various aspects of perceptions of advertising to thoroughly examine if the differences between groups may depend on the content of investigated construct (e.g., perception of persuasiveness versus food advertising knowledge). The procedures for data collection did not allow for clustering children according to their schools; therefore, the analyses of the effects of the school-level variables could not be conducted. Next, this study accounted for excessive body mass status incorporating both overweight and obese individuals whereas previous research showed that the differentiation between overweight and obesity may be relevant. For example, studies showed that parents may misperceive their children’s body mass, especially when it comes to differentiating between child being overweight or obese [46]. There is also evidence that obese children have more accurate perceptions of their body mass than overweight children [48,49]. Future studies should verify whether perceptions of availability of at-home and out-of-home environmental factors are different in parent–child dyads with overweight, compared to parent–child dyads with obesity. The sample was not representative for the general population of the country (e.g., in terms of parental education), which limits the generalizability of the findings. Any generalization to ethnically diverse populations should be made with caution as the analyzed sample was ethnically homogeneous (all participants were Caucasian).

To conclude, this is the first study to assess differences between parent–child dyads with normal body mass and dyads with excessive body mass in terms of perceptions of at-home (perceptions of the availability of healthy food at home) and out-of-home environment (perceptions of school and community promotion of healthy eating, perceptions of persuasiveness of food advertising). Future programs targeting obesity reduction may address specific perceptions of at-home and out-of-home environment, in particular when designing interventions targeting parents and children who already have excessive body weight. The perceptions of availability of healthy food at home, and perceptions of school and local community promotion of healthy eating may be relatively low in parent–child dyads with excessive weight, which in turn may constitute a risk factor for the maintenance of excessive body weight.

## Figures and Tables

**Table 1:** Differences in at-home and out-of-home environment: Comparisons of dyads of parents and children with excessive body mass (*n* = 129) and dyads of parents and children with normal body mass (*n* = 377).

	*M (SD)* for Parent–child Dyads with Excessive Body Mass/*M (SD)* for Parent–child Dyads with Normal Body Mass	Between-Groups Differences		
		*F (df)* for the Model without Covariates/*F (df)* for the Model with Covariates	*η*^2^ for the Model without Covariates/*η*^2^ for the Model with Covariates	Cohen’s *d* (95% CI)
Healthy food availability (P, T1)	2.94 (0.42)/3.08 (0.39)	13.01 (1, 504) ***/6.23 (4, 501) ***	**0.025/0.047**	0.35 (0.32, 0.39)
Healthy food availability (P, T2)	2.97 (0.33)/3.11 (0.30)	19.22 (1, 504) ***/7.74 (4, 501) ***	**0.037/0.058**	0.42 (0.39, 0.45)
School and local promotion (P, T1)	2.70 (0.66)/2.84 (0.67)	3.91 (1, 504) */2.44 (4, 501) *	**0.008/0.019**	0.21 (0.15, 0.27)
School and local promotion (P, T2)	2.73 (0.53)/2.83 (0.53)	**3.69 (1, 504) ^ⴕ^/2.96 (4, 501) ^ⴕ^**	**0.007/0.008**	0.19 (0.14, 0.24)
Advertisement perception (P, T1)	2.41 (0.98)/2.42 (0.98)	0.01 (1, 504)/0.42(4, 501)	<0.001/0.003	0.01 (−0.08, 0.10)
Advertisement perception (P, T2)	2.48 (1.10)/2.52 (1.05)	0.11 (1, 504)/0.23 (4, 501)	<0.001/0.002	0.04 (−0.06, 0.13)
Healthy food availability (Ch, T1)	2.85 (0.46)/2.84 (0.44)	0.02 (1, 504)/3.18 (4, 501) *	<0.001/0.025	−0.02 (−0.06, 0.16)
Healthy food availability (Ch, T2)	2.75 (0.37)/2.82 (0.34)	3.24 (1, 504) ^ⴕ^/5.36 (4, 501) ***	**0.006/0.041**	0.20 (0.17, 0.23)
Advertisement perception (Ch, T1)	2.54 (0.71)/2.59 (0.83)	0.34 (1, 504)/0.12 (4, 501)	0.001/0.002	0.06 (−0.01, 0.13)
Advertisement perception (Ch, T2)	2.49 (0.79)/2.52 (0.80)	0.23 (1, 504)/0.93 (4, 501)	<0.001/0.007	0.04 (−0.03, 0.11)

*** *p* < 0.001; * *p* < 0.05; ^ⴕ^ *p* < 0.10; P = parent; Ch = child; T1 = Time 1 (baseline); T2 = Time 2 (10-month follow-up); for all analyses *df* = 1, 504; Advertisement perception = perceptions of persuasiveness of food advertising; Local promotion = perceptions of school and local community promotion of healthy nutrition; Healthy food availability = perceptions of availability of heathy food at home. Covariates included: the parental education level, parental perceived economic status, and size of the place of residence. Significant differences (with both significant *p*-levels and significant 95% CI for Cohen’s *d*) are marked in bold.

## Appendix A

**Table A1:** Demographic and clinical characteristics of *N* = 798 parent–child dyads and the main analyzed sample (*N* = 506 dyads) including, parent–child dyads with excessive body mass (*n* = 129) and parent–child dyads with normal body mass (*n* = 377).

	Parent–Child Dyads (*N* = 798)		Parent–Child Dyads with Excessive Body Mass (*n* = 129)		Parent–Child Dyads with Normal Body Mass (*n* = 377)		Parents from Dyads with Excessive Body Mass vs. Parents from Dyads with Normal Body Mass			Children from Dyads with Excessive Body Mass vs. Children from Dyads with Normal Body Mass		
	% or Range (*M*; *SD*)											
	**Parent**	**Child**	**Parent**	**Child**	**Parent**	**Child**	***χ*^2^*(df)* or *F (df)***	***η*^2^**	**Cohen’s *d* (95% CI)**	***χ*^2^*(df)* or *F (df)***	***η*^2^**	**Cohen’s *d* (95% CI)**
Gender							**12.83** **(1) ****	**0.025**	**0.04** **(0.02, 0.06)**	**2.60** **(1)** **^ⴕ^**	**0.005**	**0.04** **(0.01, 0.07)**
Female	*n* = 707 (88.6%)	*n* = 433 (54.3%)	*n* = 108 (83.7%)	*n* = 80 (62.0%)	*n* = 354 (93.9%)	*n* = 203 (53.8%)						
Male	*n* = 91 (11.4%)	*n* = 365 (45.7%)	*n* = 21 (16.3%)	*n* = 49 (38.0%)	*n* = 23 (6.1%)	*n* = 174 (46.2%)						
T1 Age	23–66 (36.40; 5.38)	5–12 (7.80; 1.46)	23–49 (36.03; 5.44)	5–11 (7.86; 1.38)	24–59 (36.22; 5.01)	5–10 (7.78; 1.51)	0.14 (1, 504)	<0.001	0.04 (−0.31, 0.39)	0.27 (1, 504)	0.001	−0.06 (−0.15, 0.04)
T2 Age	23–67 (36.82; 4.24)	6–12 (8.33; 1.12)	23–49 (36.49; 5.39)	6–12 (8.41; 1.38)	24–51 (36.79; 4.99)	6–11 (8.37; 1.46)	0.21 (1, 504)	0.001	0.06 (−0.29, 0.41)	0.05 (1, 504)	<0.001	−0.03 (−0.12, 0.07)
T1 BMI	18.50–46.87 (24.92; 4.43)	13.92–33.74 (17.60; 2.79)	25.08–46.87 (29.84; 4.11)	17.50–33.74 (21.49; 2.65)	18.50–24.92 (21.88; 1.70)	13.92–19.95 (16.18; 1.27)	**945.89** **(1, 504) *****	**0.652**	**2.74** **(2.54, 2.93)**	**909.89** **(1, 504) *****	**0.644**	**2.74** **(2.61, 2.87)**
T2 BMI	17.86–42.86 (24.91; 4.27)	11.96–30.56 (17.33; 3.04)	21.93–42.87 (29.50; 3.86)	17.01–30.56 (21.30; 3.02)	17.86–27.25 (22.00; 1.76)	13.06–20.93 (16.31; 1.46)	**888.21** **(1, 504) *****	**0.638**	**2.69** **(2.50, 2.88)**	**371.57** **(1, 504) ****	**0.552**	**2.69** **(2.50, 2.88)**
Education:							**4.41** **(1, 504) ***	**0.009**	**0.21** **(0.13, 0.30)**			
Primary	*n* = 20 (2.5%)		*n* = 4 (3.1%)		*n* = 11 (2.9%)							
Vocational	*n* = 100 (125%)		*n* = 21 (16.3%)		*n* = 40 (10.6%)							
Secondary	*n* = 216 (27.1%)		*n* = 35 (27.1%)		*n* = 93 (24.7%)							
Post-secondary	*n* = 81 (10.2%)		*n* = 17 (13.2%)		*n* = 37 (9.8%)							
Higher	*n* = 381 (47.7%)		*n* = 52 (40.3%)		*n* = 195 (51.7%)							
Economic status:							**4.98** **(1, 504) ***	**0.010**	**0.22** **(0.17, 0.27)**			
Higher	*n* = 68 (8.5%)		*n* = 37 (28.7%)		*n* = 127 (33.7%)							
Similar	*n* = 483 (60.5%)		*n* = 74 (57.4%)		*n* = 224 (59.5%)							
Lower	*n* = 247 (31.0%)		*n* = 18 (14.0%)		*n* = 26 (6.9%)							
Place of residence							1.18 (1, 504)	0.002	0.11 (0.03, 0.19)			
≤10,000 residents	*n* = 244 (30.6%)		*n* = 43 (33.3%)		*n* = 99 (26.3%)							
10,000–100,000 residents	*n* = 172 (21.6%)		*n* = 26 (20.2%)		*n* = 88 (23.3%)							
100,000–500,000 residents	*n* = 114 (14.3%)		*n* = 21 (16.3%)		*n* = 67 (17.9%)							
≥500,000 residents	*n* = 268 (33.6%)		*n* = 39 (30.2%)		*n* = 123 (32.6%)							

*** *p* < 0.001; ** *p* < 0.01; * *p* < 0.05; ^ⴕ^ *p* < 0.10; T1 = Time 1 (baseline); T2 = Time 2 (10-month follow-up); BMI = body mass index; Education = the parental education level; Economic status = the parental perceived economic status (reports on comparison to the economic situations of the average family in the country). Significant differences (with significant *p* = 0.05 levels and significant 95% CI for Cohen’s *d*) are marked in bold.

**Table A2:** Correlations and descriptive statistics for the study variables: Characteristics of the main analyzed sample (*N* = 506 parent–child dyads with normal body mass and parent–child dyads with excessive body mass) and for *N* = 798 (four types of dyads: parent with excessive body mass and child with normal body mass; parent with normal body mass and child with excessive body mass; parent–child dyads with excessive body mass; parent–child dyads with normal body mass).

		*M* (*SD*) for *N* = 506 Parent–child Dyads /*M* (*SD*) for *N* = 798 Parent–child Dyads	1	2	3	4	5	6	7	8	9	10	11	12	13	14	15	16	17	18	19	20
**1**	Healthy food availability (P, T1)	3.05 (0.40)/ 3.02 (0.40)																				
**2**	Healthy food availability (P, T2)	3.07 (0.32)/ 3.04 (0.31)	**0.54/** **0.50**																			
**3**	School and local promotion (P, T1)	2.80 (0.67)/ 2.76 (0.68)	0.01/ 0.02	0.02/ 0.05																		
**4**	School and local promotion (P, T2)	2.81 (0.53)/ 2.79 (0.52)	0.03/ 0.04	**0.16/** **0.14**	**0.39/** **0.41**																	
**5**	Advertisement perception (P, T1)	2.42 (0.98)/ 2.50 (0.98)	−0.05/ −0.06	0.01/ −0.01	0.01/ −0.02	−0.04/ −0.05																
**6**	Advertisement perception (P, T2)	2.51 (1.06)/ 2.56 (0.79)	−0.07/ −0.01	−0.09/ 0.01	−0.07/ 0.03	−0.06/ −0.05	**0.39/** **0.33**															
**7**	Healthy food availability (Ch, T1)	2.84 (0.44)/ 3.03 (0.47)	**0.25/** **0.23**	**0.11/** **0.15**	0.02/ 0.01	0.02/ −0.01	−0.02/ −0.03	−0.10/ −0.06														
**8**	Healthy food availability (Ch, T2)	3.07 (0.32)/ 3.17 (0.32)	**0.29/** **0.20**	**0.39/** **0.31**	0.06/ 0.03	**0.11/** **0.12**	−0.01/ 0.03	−0.09/ −0.05	**0.32/** **0.34**													
**9**	Advertisement perception (Ch, T1)	2.57 (0.80)/ 2.49 (1.04)	−0.01/ −0.07	0.01/ −0.02	0.05/ 0.04	0.09/ 0.04	**0.30/** **0.27**	**0.11/** **0.06**	0.08/ 0.09	0.10/ 0.10												
**10**	Advertisement perception (Ch, T2)	2.51 (0.80)/ 2.53 (0.80)	−0.10/ −0.08	−0.15/ −0.10	−0.02/ −0.04	−0.01/ −0.03	−0.02/ 0.03	**0.30/** **0.24**	−0.05/ −0.04	−0.15/ −0.13	**0.13/** **0.16**											
**11**	BMI (P, T1)	23.91 (4.30)/ 24.92 (4.43)	**−0.11/** **−0.13**	**−0.17/** **−0.14**	−0.04/ −0.04	−0.06/ −0.06	−0.02/ −0.01	−0.06/ 0.01	−0.02/ −0.06	−0.06/ −0.04	−0.01/ −0.03	−0.02/ −0.02										
**12**	BMI (P, T2)	23.92 (4.10)/ 24.91 (4.27)	**−0.12/** **−0.14**	**−0.17/** **−0.16**	−0.06/ −0.04	−0.07/ −0.06	−0.03/ −0.03	−0.04/ 0.01	−0.02/ −0.06	−0.08/ −0.04	−0.01/ −0.01	−0.01/ −0.01	**0.98**/ **0.9**8									
**13**	BMI (Ch, T1)	17.53 (2.78)/ 17.60 (2.79)	**−0.12/** **−0.10**	**−0.15/** **−0.11**	−0.07/ −0.07	−0.07/ −0.06	−0.05/ **−0.0**9	−0.06/ −0.01	0.03/ 0.04	−0.08/ −0.02	−0.02/ −0.02	−0.06/ −0.02	**0.67/** **0.65**	**0.67**/ **0.65**								
**14**	BMI (Ch, T2)	17.43 (2.73)/ 17.33 (3.04)	**−0.15/** **−0.12**	**−0.21/** **−0.22**	0.02/ 0.05	−0.07/ −0.02	0.04/ −0.01	−0.08/ −0.02	−0.03/ −0.06	−0.09/ −0.05	−0.02/ −0.04	−0.09/ −0.07	**0.62/** **0.62**	**0.62/** **0.62**	**0.93/** **0.89**							
**15**	Age (P, T1)	36.17 (5.12)/ 36.40 (5.38)	**0.14/** **0.11**	**0.16/** **0.12**	−0.04/ −0.04	0.05/ 0.05	**−0.11/** **−0.07**	**−0.12/** **−0.07**	**0.12/** **0.10**	**0.10/** **0.07**	−0.01/ −0.01	**−0.12/** **−0.07**	0.01/ 0.01	0.02/ 0.01	0.07/ 0.03	0.01/ 0.03						
**16**	Age (Ch, T1)	7.80 (1.48)/ 7.80 (1.46)	0.03/ 0.01	0.01/ −0.02	−0.07/ −0.06	0.05/ 0.01	−0.05/ −0.02	**−0.09/** **−0.04**	0.01/ 0.02	0.01/ 0.04	−0.05/ −0.08	**−0.18/** **−0.14**	0.04/ −0.02	0.05/ −0.01	**0.25/** **0.27**	**0.22/** **0.13**	**0.23/** **0.20**					
**17**	Gender (P, T1)	1.91 (0.28)/ 1.89 (0.32)	0.01/ 0.06	0.05/ 0.04	0.06/ 0.06	0.05/ 0.03	0.02/ −0.01	0.02/ 0.02	−0.06/ −0.06	−0.01/ 0.04	0.02/ 0.04	−0.04/ −0.03	**−0.19/** **−0.19**	**−0.18/** **−0.18**	**−0.16/** **−0.16**	−0.08/ −0.03	**−0.11/** **−0.17**	−0.04/ −0.07				
**18**	Gender (Ch, T1)	1.56 (0.50)/ 1.54 (0.50)	0.08/ 0.05	0.06/ 0.02	−0.01/ −0.03	−0.01/ 0.01	0.05/ 0.04	−0.01/ 0.02	**0.14/** **0.07**	0.08/ 0.08	0.02/ −0.02	−0.02/ −0.02	0.07/ 0.03	0.06/ 0.03	0.03/ −0.01	0.03/ 0.05	0.06/ 0.04	0.05/ 0.02	0.02/ 0.02			
**19**	Education	3.91 (1.22)/ 3.88 (1.21)	**0.16/** **0.13**	**0.16/** **0.19**	−0.08/ −0.08	0.01/ 0.03	0.05/ 0.07	−0.02/ 0.06	**0.10/** **0.08**	**0.15/** **0.09**	0.04/ 0.07	−0.07/ −0.09	**−0.14/** **−0.13**	**−0.12/** **−0.14**	**−0.12/** **−0.09**	−0.10/ −0.02	**0.20**/ **0.19**	−0.06/ −0.02	**−0.08/** **−0.0**7	−0.01/ −0.01		
**20**	Economic status	2.70 (0.77)/ 2.72 (0.75)	0.08/ 0.08	0.04/ 0.04	−0.05/ −0.04	−0.01/ −0.02	−0.01/ −0.01	0.03/ 0.03	**0.13**/ **0.14**	**0.16/** **0.15**	−0.05/ −0.01	−0.05/ −0.09	**−0.10/** **−0.08**	**−0.10/** **−0.08**	**−0.11/** **−0.09**	**−0.11/** **−0.07**	0.04/ 0.04	−0.03/ −0.03	−0.05/ −0.04	0.02/ 0.03	**0.25/** **0.24**	
**21**	Place of residence	2.51 (1.23)/ 2.46 (1.24)	0.03/ 0.02	0.06/ 0.06	−0.05/ −0.04	0.01/ 0.02	−0.02/ 0.03	−0.01/ 0.01	−0.04/ −0.01	0.06/ 0.02	**0.11/** **0.07**	0.02/ 0.01	0.03/ 0.01	0.01/ −0.01	−0.07/ −0.08	−0.06/ −0.02	**0.11/** **0.14**	**−0.07/** **−0.08**	−0.04/ −0.04	0.02/ 0.03	**0.18/** **0.17**	−0.04/ −0.03

P = parent; Ch = child; T1 = Time 1 (baseline); T2 = Time 2 (10-month follow-up). BMI = body mass index; Advertisement perception = perception of persuasiveness of food advertising; School and local promotion = perception of school and local community promotion of healthy eating; Healthy food availability = perceptions of availability of heathy food at home; Education = the parental education level (1—primary, 2—uncompleted secondary/vocational, 3—secondary, 4—≤3 years of higher education, 5—≥4 years of higher education); Economic status = the parental perceived economic status (reports on comparison to the economic situations of the average family in the country; 1—much below the average, 2—below average, 3—similar to average, 4—above the average, 5—much above the average); Place of residence (1—<10,000 inhabitants, 2—between 10,000 and 100,000 inhabitants, 3—between 100,000 and 500,000 inhabitants, 4—>500,000 inhabitants); Gender (1—male; 2—female). Person’s *r* for continuous variables and Spearman’s *rho* for categorical variables are provided. Significant (at *p* < 0.05) coefficients are marked in bold.

**Table A3:** Differences in perceptions of at-home and out-of-home environment: Parent–child dyads with excessive body mass (*n* = 129) versus parent–child dyads with normal body mass (*n* = 377).

		*M* (*SD*) for Parent–child Dyads with Normal Body Mass at T1 → at T2/*M* (*SD*) for Parent–child Dyads with Excessive Body Mass at T1 → at T2	Time Effects (T1 Variable → T2 Variable)						Interaction Effects (Time*Group)	
			Parent–child Dyads with Excessive Body Mass			Parent–child Dyads with Normal Body Mass			*F* for the Model without Covariates/*F* for the Model with Covariates	*η*^2^ for the Model without Covariates/*η*^2^ for the Model with Covariates
			*F* for the Model without Covariates/*F* for the Model with Covariates	*η*^2^ for the Model without Covariates/*η*^2^ for the Model with Covariates	Cohen’s *d* (95% CI)	*F* for the Model without Covariates/*F* for the Model with Covariates	*η*^2^ for the Model without Covariates/*η*^2^ for the Model with Covariates	Cohen’s *d* (95% CI)		
1	Healthy food availability (P, T1) → Healthy food availability (P, T2)	3.08 (0.39) → 3.11 (0.30)/2.94 (0.42) → 2.97 (0.33)	0.96/<0.01	0.007/<0.001	0.08 (0.04, 0.11)	1.70/0.02	0.005/<0.001	0.08 (0.05, 0.11)	0.26/0.01	< 0.001/<0.001
2	School and local promotion (P, T1) → School and local promotion (P, T2)	2.84 (0.67) → 2.83 (0.53)/2.70 (0.66) → 2.73 (0.53)	0.25/0.39	0.002/0.003	0.05 (−0.10, 0.01)	0.01/0.44	<0.001/0.001	0.02 (−0.04, 0.07)	0.20/0.52	<0.001/0.001
3	Advertisement perception (P, T1) → Advertisement perception (P, T2)	2.42 (0.98) → 2.52 (1.05)/2.41 (0.98) → 2.48 (1.10)	0.29/<0.01	0.002/<0.001	0.07 (−0.16, 0.02)	1.91/2.43	0.005/0.006	0.10 (0.01, 0.19)	0.03/0.05	<0.001/<0.001
4	Healthy food availability (Ch, T1) → Healthy food availability (Ch, T2)	2.84 (0.44) → 2.82 (0.34)/2.85 (0.46) → 2.75 (0.37)	2.28/2.59	0.019/0.023	0.23 (0.19, 0.27)	0.98/0.06	0.003/<0.001	0.05 (0.01, 0.08)	2.19/2.01	0.004/0.004
5	Advertisement perception (Ch, T1) → Advertisement perception (Ch, T2)	2.59 (0.83) → 2.52 (0.80)/2.54 (0.71) → 2.49 (0.79)	0.31/0.13	0.002/0.001	0.07 (0.01, 0.13)	1.25/1.33	0.003/0.007	0.09 (0.01, 0.16)	0.01/0.01	<0.001/<0.001

All *F* values reported in this table are not significant, *p*s > 0.05; P = parent; Ch = child; T1 = time 1 (baseline); T2 = time 2 (10-month follow-up); Advertisement perception = perceptions of persuasiveness of food advertising; Local promotion = perceptions of school and local community promotion of healthy eating; Healthy food availability = perceptions of availability of heathy food at home. Covariates included parental education level, parental perceived economic status and place of residence.

**Table A4:** Differences in the study variables and demographic variables between excessive body mass parent-normal body mass child dyads (*n* = 193), normal body mass parent-excessive body mass child dyads (*n* = 88), parent–child dyads with excessive body mass (*n* = 129), and parent–child dyads with normal body mass (*n* = 377).

	*M (SD)* for (1) Excessive Body Mass Parent-Normal Body Mass Child Dyads/*M (SD)* for (2) Normal Body Mass Parent-Excessive Body Mass Child Dyads/*M (SD)* for (3) parent–child Dyads with Excessive Body Mass/*M (SD)* for (4) Parent–child Dyads with Normal Body Mass	Between Groups Differences		(1) vs. (2)		(1) vs. (3)		(1) vs. (4)		(2) vs. (3)		(2) vs. (4)		(3) vs. (4)	
		*F (df)* or *χ*^2^ *(df)*	*η* ^2^	Cohen’s *d* (95% CI)	Post-hoc THSD *p* (95% CI)	Cohen’s *d* (95% CI)	Post-hoc THSD *p* (95% CI)	Cohen’s *d* (95% CI)	Post-hoc THSD *p* (95% CI)	Cohen’s *d* (95% CI)	Post-hoc THSD *p* (95% CI)	Cohen’s *d* (95% CI)	Post-hoc THSD *p* (95% CI)	Cohen’s *d* (95% CI)	Post-hoc THSD *p* (95% CI)
Healthy food availability (P, T1)	2.94 (0.41)/3.05 (0.34)/2.94 (0.42)/3.08 (0.39)	**7.80** **(3, 787) *****	**0.029**	NS		NS		**−0.35** **(−0.39, −0.32)**	**<0.001** **(−0.23, −0.05)**	NS		NS		**0.35** **(0.32, 0.39)**	**0.002** **(0.04, 0.25)**
Healthy food availability (P, T2)	3.00 (0.31)/2.99 (0.30)/2.97 (0.33)/3.11 (0.30)	**8.51** **(3, 787) *****	**0.031**	NS		NS		**−0.36** **(−0.39, −0.34)**	**0.006** **(−0.16, −0.02)**	NS		NS		**0.42** **(0.39, 0.45)**	**<0.001** **(0.06, 0.22)**
School and local promotion (P, T1)	2.70 (0.69)/2.67 (0.66)/2.70 (0.66)/2.84 (0.67)	**2.97** **(3, 787) ***	**0.011**	NS		NS		NS		NS		NS		**0.21** **(0.15, 0.27)**	**0.024** **(0.02, 0.31)**
School and local promotion (P, T2)	2.75 (0.53)/2.83 (0.43)/2.73 (0.53)/2.83 (0.53)	1.95 (3, 787)	0.007	NS		NS		NS		NS		NS		0.19 (0.14, 0.24)	**0.032** **(0.04, 0.42)**
Advertisement perception (P, T1)	2.62 (0.97)/2.66 (1.01)/2.41 (0.98)/2.42 (0.98)	3.03 (3, 787)	0.001	NS		NS		NS		NS		NS		NS	
Advertisement perception (P, T2)	2.55 (0.78)/2.58 (0.70)/2.48 (1.10)/2.52 (1.05)	0.15 (3, 787)	0.001	NS		NS		NS		NS		NS		NS	
Healthy food availability (Ch, T1)	2.84 (0.45)/2.95 (0.47)/2.85 (0.46)/2.84 (0.44)	1.70 (3, 787)	0.005	NS		NS		NS		NS		NS		NS	
Healthy food availability (Ch, T2)	2.81 (0.44)/2.97 (0.40)/2.75 (0.37)/2.82 (0.34)	**3.50** **(3, 787) ^ⴕ^**	**0.009**	NS		NS		NS		NS		NS		**0.20** **(0.17, 0.23)**	**0.039** **(0.02, 0.24)**
Advertisement perception (Ch, T1)	2.42 (0.99)/2.57 (1.05)/2.54 (0.71)/2.59 (0.83)	0.54 (3, 787)	0.002	NS		NS		NS		NS		NS		NS	
Advertisement perception (Ch, T2)	2.53 (0.84)/2.60 (0.68)/2.49 (0.79)/2.52 (0.80)	0.34 (3, 787)	0.001	NS		NS		NS		NS		NS		NS	
Gender (P, T1)	1.79 (0.41)/1.96 (0.21)/1.84 (0.37)/1.94 (0.24)	**12.82** **(3)**	**0.046**	**−0.47** **(−0.52, −0.43)**	**<0.001** **(−0.27, −0.07)**	NS		**−0.49** **(−0.51, −0.46)**	**<0.001** **(−0.22, −0.08)**	**0.38** **(0.34, 0.42)**	**0.031** **(0.01, 0.23)**	NS		**0.04** **(0.02, 0.06)**	**0.008** **(0.02, 0.18)**
Age (P, T1)	38.01 (5.81)/35.41 (5.48)/36.03 (5.44)/36.22 (5.01)	1.56 (3, 787)	0.002	NS		NS		NS		NS		NS		NS	
BMI (P, T1)	28.69 (3.14)/22.11 (1.71)/29.84 (4.11)/21.88 (1.70)	**491.45** **(3, 787) *****	**0.650**	**2.38** **(2.06, 2.70)**	**<0.001** **(5.72, 7.44)**	NS		**2.98** **(2.79, 3.17)**	**<0.001** **(6.22, 7.40)**	**−2.32** **(−2.76, −1.87)**	**<0.001** **(−8.67, −6.80)**	NS		**2.74** **(2.54, 2.93)**	**<0.001** **(7.28, 8.66)**
BMI (P, T2)	28.55 (3.08)/22.26 (1.96)/29.50 (3.86)/22.00 (1.76)	**458.07** **(3, 787) *****	**0.634**	**2.27** **(1.95, 2.60)**	**<0.001** **(5.44, 7.16)**	NS		**2.86** **(2.67, 3.05)**	**<0.001** **(5.97, 7.13)**	**−2.25** **(−2.68, −1.83)**	**<0.001** **(−8.16, −6.32)**	NS		**2.69** **(2.50, 2.88)**	**<0.001 (6.82, 8.18)**
Education (P, T1)	3.83 (1.20)/3.85 (1.19)/3.71 (1.24)/3.97 (1.21)	1.72 (3, 787)	0.006	NS		NS		NS		NS		NS		NS	
Economic status (P, T1)	2.77 (0.75)/2.73 (0.65)/2.83 (0.74)/2.66 (0.78)	2.10 (3, 787)	0.008	NS		NS		NS		NS		NS		NS	
Place of residence (P, T1)	2.37 (1.27)/2.35 (1.24)/2.41 (1.26)/2.55 (1.22)	1.20 (3, 787)	0.005	NS		NS		NS		NS		NS		NS	
Gender (Ch)	1.51 (0.50)/1.51 (0.50)/1.62 (0.49)/1.54 (0.50)	1.39 (3)	0.005	NS		NS		NS		NS		NS		NS	
Age (Ch, T1)	7.79 (1.42)/7.82 (1.29)/7.80 (1.48)/7.86 (1.38)	0.37 (3, 787)	0.001	NS		NS		NS		NS		NS		NS	
BMI (Ch, T1)	16.42 (1.44)/20.71 (2.22)/21.49 (2.65)/16.18 (1.27)	**432.61** **(3, 787) *****	**0.621**	**−2.70** **(−2.70, −2.30)**	**<0.001** **(−4.86, −3.72)**	**−2.53** **(−2.75, −2.31)**	**<0.001** **(−5.67, −4.57)**	NS		NS		**3.04** **(2.90, 3.17)**	**<0.001** **(4.01, 5.06)**	**2.74** **(2.61, 2.87)**	**<0.001 (4.86, 5.77)**
BMI (Ch, T2)	16.88 (1.51)/20.62 (3.01)/21.30 (3.02)/16.31 (1.46)	**418.16** **(3, 787) *****	**0.549**	**−1.79** **(−2.04, −1.55)**	**<0.001** **(−3.25, −0.74)**	**−1.98** **(−2.22, −1.74)**	**0.042** **(−2.21, −0.03)**	NS		NS		**2.33** **(2.16, 2.50)**	**<0.001** **(0.73, 3.08)**	**2.69** **(2.50, 2.88)**	**0.039 (0.03, 2.02)**

*** *p* < 0.001; * *p* < 0.05; ^ⴕ^ *p* < 0.10; T1 = Time 1 (baseline); T2 = Time 2 (10-month follow-up); BMI = body mass index; Education = parental education level; Economic status = parental perceived economic status (reports on comparison to the economic situations of the average family in the country). Cohen’s *d* is provided only for significant between groups differences. Significant differences (*p* < 0.05 and significant 95% CI for Cohen’s *d*) are marked in bold.
